# Partitioning global change: Assessing the relative importance of changes in climate and land cover for changes in avian distribution

**DOI:** 10.1002/ece3.4890

**Published:** 2019-01-30

**Authors:** Matthew J. Clement, James D. Nichols, Jaime A. Collazo, Adam J. Terando, James E. Hines, Steven G. Williams

**Affiliations:** ^1^ United States Geological Survey Patuxent Wildlife Research Center Laurel Maryland; ^2^ Arizona Game and Fish Department, Research Branch Phoenix Arizona; ^3^ United States Geological Survey, North Carolina Cooperative Fish and Wildlife Research Unit, Department of Applied Ecology North Carolina State University Raleigh North Carolina; ^4^ Southeast Climate Adaptation Science Center U.S. Geological Survey Raleigh North Carolina; ^5^ North Carolina Cooperative Fish and Wildlife Research Unit, Department of Applied Ecology North Carolina State University Raleigh North Carolina

**Keywords:** dynamic occupancy models, Eastern Wood Pewee, local colonization, local extinction, North American Breeding Bird Survey, Red‐eyed Vireo

## Abstract

Understanding the relative impact of climate change and land cover change on changes in avian distribution has implications for the future course of avian distributions and appropriate management strategies. Due to the dynamic nature of climate change, our goal was to investigate the processes that shape species distributions, rather than the current distributional patterns. To this end, we analyzed changes in the distribution of Eastern Wood Pewees (*Contopus virens*) and Red‐eyed Vireos (*Vireo olivaceus*) from 1997 to 2012 using Breeding Bird Survey data and dynamic correlated‐detection occupancy models. We estimated the local colonization and extinction rates of these species in relation to changes in climate (hours of extreme temperature) and changes in land cover (amount of nesting habitat). We fit six nested models to partition the deviance explained by spatial and temporal components of land cover and climate. We isolated the temporal components of environmental variables because this is the essence of global change. For both species, model fit was significantly improved when we modeled vital rates as a function of spatial variation in climate and land cover. Model fit improved only marginally when we added temporal variation in climate and land cover to the model. Temporal variation in climate explained more deviance than temporal variation in land cover, although both combined only explained 20% (Eastern Wood Pewee) and 6% (Red‐eyed Vireo) of temporal variation in vital rates. Our results showing a significant correlation between initial occupancy and environmental covariates are consistent with biological expectation and previous studies. The weak correlation between vital rates and temporal changes in covariates indicated that we have yet to identify the most relevant components of global change influencing the distributions of these species and, more importantly, that spatially significant covariates are not necessarily driving temporal shifts in avian distributions.

## 1 INTRODUCTION

In recent decades, the distributions of many bird species have changed in Britain (Fuller et al., 1995; Thomas & Lennon, 1999), continental Europe (Böhning‐Gaese & Bauer, 1996; Brommer, Lehikoinen, & Valkama, 2012), South Africa (Hockey, Sirami, Ridley, Midgley, & Babiker, 2011), and North America (Sauer, Link, Fallon, Pardieck, & Ziolkowski, 2013). These shifts are of both ecological and conservation interest, and they have stimulated a great deal of research. While various factors may be contributing to distributional shifts, including invasive species (Crowl, Crist, Parmenter, Belovsky, & Lugo, 2008), pollution (Trathan et al., 2015), exploitation (Laursen & Frikke, 2008) and other causes, climate change and land cover changes are likely to be two of the strongest drivers for many species (Barbet‐Massin, Thuiller, & Jiguet, 2012; Lemoine, Bauer, Peintinger, & Böhning‐Gaese, 2007; Travis, 2003). Understanding the relative impact of these drivers of distributional change has implications for ecological understanding, predictions of future changes, appropriate management strategies, and the allocation of conservation resources. However, species distribution models focused on the effects of climate are far more common than analyses that compare the contributions of climate and land cover (Sirami et al., 2017).

Ecologists have long debated the factors that affect species ranges (Grinnell, 1917; MacArthur, 1972) because of the relevance to ecology, biogeography, and community ecology. In recent decades, global change has given more urgency to the topic (Urban, 2015), and the expectation that both the global environment and species distributions will continue to change has stimulated much research into predicting the future course of change (Huntley et al., 2006). Questions about the relative impact of climate change and land cover change are important because of the implications for these predictions (Sirami et al., 2017). For example, climate change is expected to occur along a latitudinal gradient, while changes in land cover may exhibit less directionality if factors such as urbanization or conversion to or from agricultural use are significant drivers of land cover change (Lemoine et al., 2007; Travis, 2003). As a result, species distributions that respond primarily to climate are more likely to also exhibit directionality (Brommer et al., 2012; Hockey et al., 2011; Parmesan & Yohe, 2003). Whether species distributions respond to land cover or climate change has implications for appropriate responses, such as the design and location of reserves (Araújo, Cabeza, Thuiller, Hannah, & Williams, 2004), habitat restoration, and assisted migration (Hoegh‐Guldberg et al., 2008). Accordingly, politically and economically significant decisions may be influenced by these projections. Therefore, there is a need for research into the relative effects of climate change and land cover change on changes in the distribution of species.

When there is interest in projecting changes in species distributions, a crucial insight is that environmental factors that correlate with the current species distribution may not correlate with future changes in distribution (Barbet‐Massin et al., 2012). One reason is that projections developed from current distribution patterns assume that species are in equilibrium with their environment (Elith, Kearney, & Phillips, 2010), which may not be true (Zhu, Woodall, & Clark, 2012). A second reason is that dispersal limitations may prevent species from occupying their preferred habitat in the future (Devictor, Julliard, Couvet, & Jiguet, 2008). Therefore, investigating the processes that shape species distributions, rather than current species distribution patterns, is likely to generate greater ecological understanding and better projected distributions (Yackulic, Nichols, Reid, & Der, 2015). For example, the recent distribution of breeding Louisiana Waterthrush (*Parkesia motacilla*) in North America is best described by mean temperature, but changes in that distribution correlate with mean precipitation as well as mean temperature, indicating that the process cannot be fully described by the pattern observed at one point in time (Clement, Hines, Nichols, Pardieck, & Ziolkowski, 2016). We further note that data on environmental variables typically include both spatial and temporal components. For example, temperature may vary along a north–south gradient, as well as through time. When the primary research interest is in temporal changes in distributions, it makes sense to isolate the temporal components of environmental variables (which are the essence of global change) when investigating species response to global change.

Here, we assess the relative importance of changes in climate and land cover to changes in distributions for two bird species, the Red‐eyed Vireo (*Vireo olivaceus*) and the Eastern Wood Pewee (*Contopus virens*). We selected just two species with similar biology, but differing population trends, so that we could develop species‐specific hypotheses about distributional shifts. We partitioned the spatial and temporal components of our environmental predictors to improve our inferences about the processes affecting species distributions and about the likely consequences of future global change. We used dynamic occupancy modeling to explicitly estimate the vital rates that govern changes in distributions so as to avoid the equilibrium assumption implicit in static species distribution models. Finally, we used an analysis of deviance approach to assess the relative importance of climate and land cover to the changes in distributions for these two species.

## 2 MATERIALS AND METHODS

### 2.1 Hypotheses

Our goal was to assess the relative importance of climate and land cover to changes in the distributions of two bird species, the Red‐eyed Vireo and the Eastern Wood Pewee. We selected these birds because they are migratory, insectivorous, and inhabit similar plant communities (Hamel, 1992), but exhibit divergent trends in relative abundance reported by the BBS (Sauer et al., 2015). The Red‐eyed Vireo breeds in the eastern and northern United States and much of southern Canada and winters in South America. It is a common and widespread species that uses a wide variety of forest habitats to nest and glean insects from foliage. Counts of Red‐eyed Vireos have been increasing by 0.8% annually since 1966 and by 1.0% annually since 2003, with some localized declines in Florida, Texas, and the Pacific Northwest (Sauer et al., 2015). The Eastern Wood Pewee breeds in the eastern United States and parts of southeastern Canada. In contrast to Red‐eyed Vireos, counts of the Eastern Wood Pewee have been declining by 1.5% since 1966 and by 1.2% since 2003 (Sauer et al., 2015). Despite the generally negative trend, there have been localized increases in counts in the Midwest.

To estimate the relative importance of climate and land cover to changes in bird distributions, we generated testable hypotheses expressing relationships between birds and environmental covariates. A common approach is to generate an extensive set of hypotheses from a suite of environmental metrics. We can then fit the specified models and observe which metrics yield significant results. However, testing many statistical hypotheses has a propensity to identify spurious relationships and such analyses should be considered exploratory (Anderson, Burnham, Gould, & Cherry, 2001; Ioannidis, 2005). In this study, we developed two a priori hypotheses from ecological principles:

1. Changes in the total annual periods of extreme temperature drive changes in bird distributions. We focus on extreme temperature because we expect these are the periods of greatest stress due to cold or heat stress, increased metabolic costs, reduced resource availability, or other mechanisms (Dawson & Whittow, 2000; Root, 1988). We defined “extreme” relative to the thermoneutral zone (TNZ), which is the range of ambient temperatures under which endotherms can maintain their body temperature without deviating from their basal metabolic rate (Calder & King, 1974). The TNZ varies among species, but has been estimated as 18–38°C for a “generic” bird (Calder & King, 1974). Several small, temperate‐zone passerines (similar to our study species), including the Northern Cardinal, Verdin, House Sparrow, and Red‐breasted Nuthatch, have been documented to have TNZs similar to that of this “generic” bird (Dawson, 1958; Goldstein, 1974; Hinds & Calder, 1973; Khaliq, Hof, Prinzinger, Böhning‐Gaese, & Pfenninger, 2014). Therefore, we expected temperatures below 18°C and above 38°C to be associated with changes in bird distributions.
2. Changes in the amount of appropriate habitat locally available during the breeding season drive changes in bird distributions because inappropriate habitat will not provide sufficient food and shelter for breeding birds (Friggens & Finch, 2015; Iglecia, Collazo, & McKerrow, 2012). For the Eastern Wood Pewee, we defined appropriate habitats as deciduous forest, evergreen forest, and mixed forest (Hamel, 1992). For the Red‐eyed Vireo, we defined appropriate habitats as the same forest types and shrub–scrub (Hamel, 1992).

Of course, these hypotheses are not exhaustive, but we selected them as logical, general mechanisms that might underlie avian responses to climate and land cover change. We expected the paucity and specificity of our hypotheses would reduce our chances of obtaining statistically significant results by chance. For example, a null hypothesis that bird distributions will not expand at a specific temperature is harder to reject than the null hypothesis that bird distributions will not expand at any temperature. We view a more specific hypothesis as a more powerful discriminatory tool (Platt, 1964; Popper, 1959), more in keeping with the hypothetico‐deductive method (Chamberlin, 1890; Romesburg, 1981) and with a Type 1 error rate closer to the nominal level (Anderson et al., 2001; Ioannidis, 2005).

We stated our hypothesized effects in terms of *changes* in bird distribution (i.e., birds will increase where habitat is available) rather than static *patterns* of bird distribution (i.e., birds will be located where habitat is available). We prefer this formulation because it focuses on the ecological processes that underlie species distributions and their dynamics. In metapopulation theory, the vital rates describing these processes are often the local colonization and extinction rates. Models that account for ecological vital rates (i.e., dynamic models) are useful because they are relatively mechanistic, thereby strengthening the generality of findings and contributing more to ecological understanding. In contrast, in order to be useful for forecasting and projection, relationships between environmental covariates and occurrence require an assumption that the species in question is in equilibrium with the environmental factors under study (Elith et al., 2010). The more phenomenological orientation of models that focus on occurrence can be limiting when projecting model results into novel situations, such as future climate and land cover conditions (Cuddington et al., 2013; Gustafson, 2013). In contrast, dynamic models avoid the equilibrium assumption, enabling more robust predictions of occurrence under future conditions (Clement et al., 2016; Yackulic et al., 2015).

Both climate and land cover vary spatially and temporally, and each process could impact bird distributions. It is possible for the level of correlation with bird vital rates to differ across those two dimensions. For example, consider an environmental variable that is completely static through a given time period and a species distribution that shifts during the same time period. In this case, it is not possible for change in the static variable to have caused the distributional shift, because no such change occurred. However, it is still possible for colonization and extinction to correlate with the environmental variable because of a spatial correlation between them. Here, our goal was to incorporate temporal variation in covariate values into our analysis because much research into global change is motivated by interest in temporal changes in species distributions. Therefore, our hypotheses and models are structured to isolate and evaluate the ability of environmental covariates to account for spatial and temporal variation in local colonization and extinction probabilities.

### 2.2 Data

We obtained data on the detection and nondetection of breeding Red‐eyed Vireos and Eastern Wood Pewees from the North American Breeding Bird Survey (Pardieck, Ziolkowski, & Hudson, 2015; www.pwrc.usgs.gov/BBS/RawData/). The Breeding Bird Survey (BBS) is a joint project of the U.S. Geological Survey and the Canadian Wildlife Service, designed to monitor the status and trends of birds breeding in North America (Sauer et al., 2013). Since its inception in 1966, the BBS has expanded to include over 5,000 survey routes across the United States and southern Canada, approximately 3,000 of which are surveyed each year. Each route is surveyed once per year by a skilled volunteer. The date of the survey is timed to occur during peak territorial behavior, typically late May to early July, depending on latitude. Surveyors follow a prescribed route along secondary roads, stopping at approximately 800‐m intervals, and performing 3‐min roadside point counts, generating 50 counts per 39.4 km route. We selected BBS data because their great geographic and temporal extent is suitable for investigating distributional changes.

We selected a subset of the available BBS data for use in our analysis. Our modeling approach, described below, makes use of stop‐specific survey results, but these detailed results are currently available in digital formats only since 1997. Therefore, we used data from 1997 to 2012. Because our study species are found in forested regions of eastern North America, we delineated the study area by aggregating three Omernik Level I classes representing eastern forest ecoregions: Northern Forests, Eastern Temperate Forests, and Tropical Wet Forests (Commission for Environmental Cooperation, 1997; Omernik, 1995, 2004; Omernik & Griffith, 2014). This study area includes the United States east of the Great Plains (2.9 million km^2^). This region encompassed the entire breeding range of the Eastern Wood Pewee, except some areas in Canada not covered by our climate and habitat covariates. We selected BBS routes entirely within this study area for analysis. We excluded routes that were not classified as “active” by the BBS and routes that differed from the recommended length (39.4 km) by more than 25%. We only analyzed surveys conducted under acceptable conditions (i.e., acceptable weather, date, time of day, stops; reported by BBS as “runtype = 1”). On the rare occasions that a route had multiple acceptable surveys per year, we used data from the first acceptable survey. This yielded 1,371 routes for our analysis. Finally, we converted counts of birds at each stop along each BBS route to detection/nondetection data suitable for presence–absence modeling. This conversion sacrificed data richness, but allowed us to account for imperfect detection of species. Using the full count data typically requires additional assumptions (Illán et al., 2014) or incorporation of additional data sources (Hooten, Wikle, Dorazio, & Royle, 2007).

Climate data were obtained from the PRISM project (Daly et al., 2008). The PRISM output uses locally weighted regression models to interpolate long‐term weather station observations to a 4 km resolution grid. Daily interpolated climate data are available from 1981 to the present. From these data, we calculated two temperature‐based indices based on our first hypothesis, described above. The first index was calculated as the total annual hours above 38°C, while the second index was calculated as the total annual hours below 18°C.

**Figure 1:**
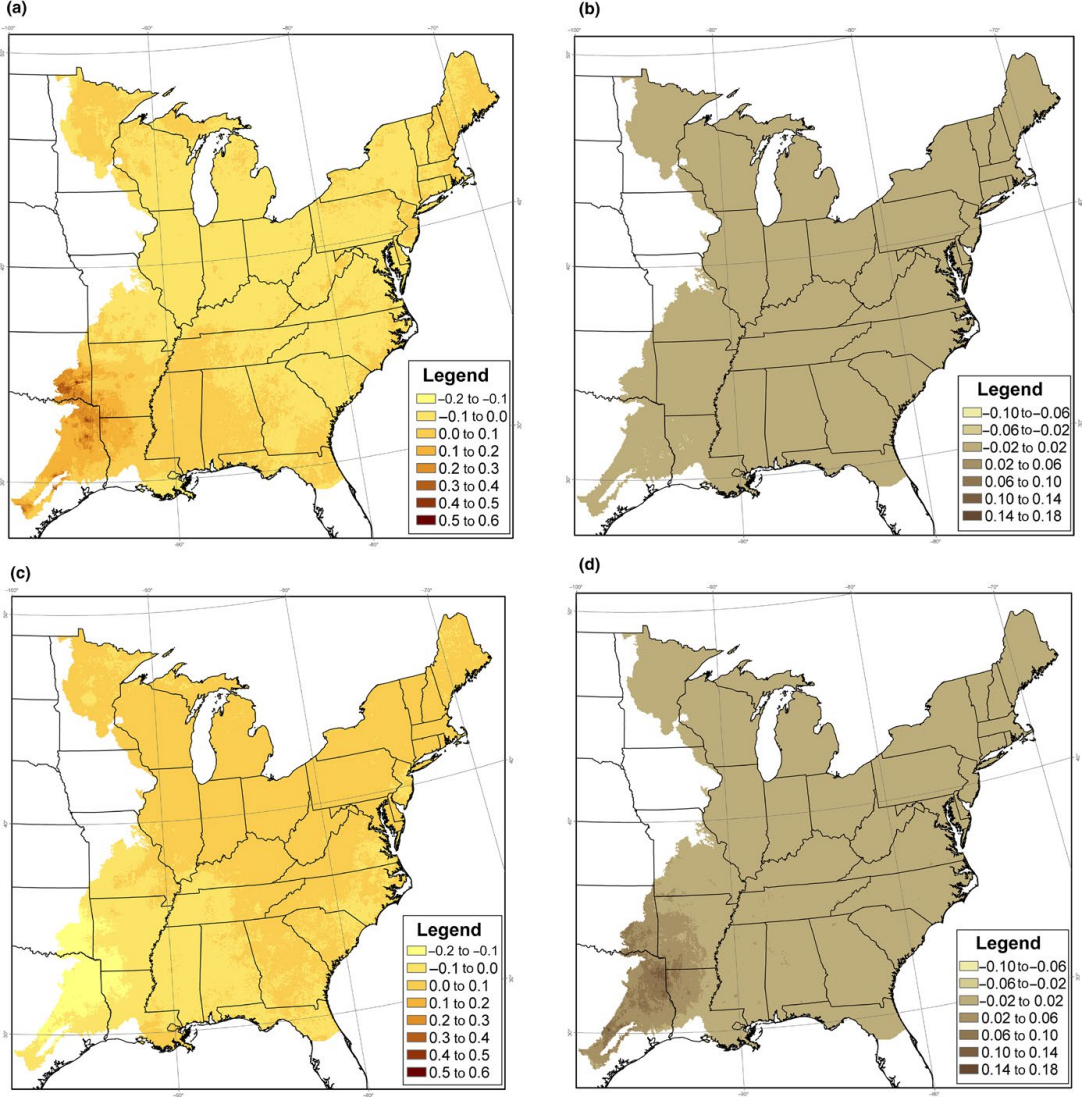
Annual deviation of colonization and extinction rates from average colonization and extinction rates$\left(\hat{\gamma }\left(t\right)-\bar{\gamma },\hat{\varepsilon }(t)-\bar{\varepsilon }\right)$, for Eastern Wood Pewee in the eastern United States, following 1997 and 2011, under Model 6. Deviations in colonization and extinction rates vary with deviations in the amount of suitable habitat, hours of heat stress, hours of cold stress, relative to average levels over the 1997–2012 study period. (a) Colonization deviation following 1997, (b) extinction deviation following 1997, (c) colonization deviation following 2011, and (d) extinction deviation following 2011

Because the temperature‐based indices were based on hours of exposure, it was necessary to interpolate the daily PRISM data to estimate hourly values. We considered a simple linear interpolation to impute values between daily maximum and minimum temperatures. However, for extreme temperatures, this method would likely result in estimated exposure durations that are biased low. Instead, we adopted the method described by Cesaraccio, Spano, Duce, and Snyder (2001; see Figure 1) and Eccel (2010) using the R software package “Interpol.T” (Eccel & Cordano, 2013). In this method, candidate functions are fit to observed hourly temperature data from nearby high‐quality ASOS (Automated Station Observing System) weather stations in the study region using a combination of sinusoidal and quadratic functions. For each 4 km PRISM grid cell, the resulting calibration function from the nearest ASOS station is then applied to each day's maximum and minimum temperature to predict the intervening hourly values.

Using the interpolated hourly temperature estimates, we calculated the total annual hours within the zones of heat stress or cold stress for each year from 1981 to 2012. Annual values were calculated over the period June 1–May 31 to represent the breeding cycle. Once the annual residence times were obtained, we applied a 15‐year moving average window to the data, resulting in 16 moving average periods starting with the 1981/82–1995/96 period and ending with the 1996/97–2011/12 period. Applying a moving average acts as a high‐pass filter that attenuates interannual variability while amplifying any long‐term climate change signals. Other period lengths could have been chosen; however, we hypothesized that 15 years represented a reasonable tradeoff between (a) reducing the moving average length so as not to exceed the length of the daily PRISM data series, which begins in 1981, (b) increasing the moving average length so that climatic changes (as opposed to background climatic variation) can be identified, and (c) using a period that is biologically meaningful in that we can reasonably expect any long‐term climatic changes in the moving averages would be outside the historical range of variability and thus should affect colonization and extinction rates of sensitive species.

We obtained land cover data from the National Land Cover Database (NLCD) for 2001 (Homer et al., 2007), 2006 (Fry et al., 2011), and 2011 (Homer et al., 2015). We reclassified the given land cover types into habitat or nonhabitat for each bird species, based on our second hypothesis, described above. We calculated the percent of land area that was a suitable habitat within 400 m of each route and used this as a covariate in our analysis. We used 400 m because this is half the distance between stops on BBS routes. For years without land cover data, we used the land cover values of the subsequent NLCD survey. Specifically, we used 2001 values for 1997–2001, 2006 values for 2002–2006, and 2011 values for 2007–2012. We used a *z*‐transformation to center and scale all covariates prior to analysis.

### 2.3 Analysis

Our goal was to assess the relative importance of climate and land cover to changes in the distribution of Red‐eyed Vireos and Eastern Wood Pewees. While our bird data consisted of detection/nondetection data from BBS surveys, our parameters of interest were the probabilities of local colonization and local extinction, as these processes drive changes in distribution. Our predictor variables were our measures of climate and land cover suitability for each species, described above. We used dynamic correlated‐detection occupancy models with detection heterogeneity to estimate the relationship between our predictors and bird distribution dynamics (Clement et al., 2016; Hines, Nichols, & Collazo, 2014; Hines et al., 2010). Occupancy models are a class of models that estimate the probability of presence while accounting for imperfect detection of species by analyzing replicated (usually temporally) surveys (MacKenzie et al., 2002, 2018). Dynamic occupancy models model interannual changes in occupancy by positing a Markov process in which the probability of occurrence at time *t* is a function of the probability of occurrence at time *t*−1 (MacKenzie, Nichols, Hines, Knutson, & Franklin, 2003). In contrast to static models, this Markov process recognizes that the distribution of a species is a function of previous environmental conditions and dispersal constraints, as well as current environmental conditions (Dormann et al., 2012; Kéry, Guillera‐Arroita, & Lahoz‐Monfort, 2013). Dynamic correlated‐detection occupancy models are a model extension in which spatially correlated replicated surveys are used to estimate detection probabilities (Hines et al., 2014, 2010). We selected an occupancy modeling approach because failure to account for imperfect detection can bias estimates of colonization and extinction rates (Ruiz‐Gutiérrez & Zipkin, 2011). We selected a dynamic approach because dynamic models offer more ecological realism, more accurate projections, and greater generalizability than static models (Clement et al., 2016; Yackulic et al., 2015). We used correlated‐detection models because the BBS generates spatially replicated surveys, and failure to account for spatial correlation of replicates can bias occupancy estimates (Hines et al., 2014, 2010). Finally, we used a finite mixture model to account for detection heterogeneity because of the variation in habitat and focal species abundance across the study area (Clement et al., 2016). A finite mixture model approximates the heterogeneity of detection probabilities by positing that the population consists of a mixture of routes which have either a relatively high detection probability or a relatively low detection probability.

As detailed above, BBS data consist of numerous routes, each composed of bird observations at 50 stops. A species may be absent from individual stops when it is present on a route, but not vice versa. Detection of a species is taken as proof of presence, but a nondetection may result from a true absence or a false absence. For clarity, we use the term “occupied” to indicate a species is present on a route and the term “available” to indicate a species is locally present at a specific stop at the time of the survey (Nichols, Thomas, & Conn, 2009). By this terminology, the dynamic correlated‐detection occupancy model includes the following parameters:

*ψ* = Pr (route occupied during the first season of surveys);
*θ = Pr (species available at stop | route occupied and species unavailable at previous stop)*;
*θ′= Pr (species available at stop | route occupied and species available at previous stop)*;
*p1 = Pr (detection at a stop for low‐detection routes | route occupied and species available at stop)*;
*p2 = Pr (detection at a stop for high‐detection routes | route occupied and species available at stop)*;
*π = Pr (probability that a route is a low‐detection route)*;
*ε_t_ = Pr (route is not occupied in season t + 1 | occupied in season t); and*
*γ_t_ = Pr (route is occupied in season t + 1 | not occupied in season t)*.

Hines et al. (2014, 2010) developed a model likelihood that allows these parameters to be modeled as functions of route‐ and year‐specific covariates, while detection parameters can also be influenced by stop‐specific covariates. Parameters can then be estimated using maximum‐likelihood estimation in program PRESENCE (Hines, 2006).

We developed six specific models of *γ* and *ε* for each bird species. We partitioned spatial and temporal variation in covariates by partitioning covariates into the average value over time, and the annual deviation from that average. In this way, the averaged covariate, *x_i_*, included spatial variation only, while the deviation component, Δ*x_iy_*, included temporal variation only. Using these covariates, our models included (a) a null model in which *γ* and *ε* are constant through time and space and (b) a global (most general) model in which *γ* and *ε* vary through space according to mean climate and land cover suitability, and through time using a dummy covariate for each year. We also considered intermediate models to assess the explanatory power of our covariates: (c) *γ* and *ε* vary spatially with mean climate and land cover suitability, but not with time, (d) *γ* and *ε* vary spatially with mean climate and land cover suitability, and temporally with annual changes in climate only, (e) *γ* and *ε* vary spatially with mean climate and land cover suitability, and temporally with annual changes in land cover only, and (f) *γ* and *ε* vary spatially with mean climate and land cover suitability, and temporally with annual changes in climate and land cover.

Prior to comparing these models, we worked to achieve adequate fit for the other model parameters. Because of the number of model parameters, we considered them sequentially. Initially, we fit the global model for *γ* and *ε*, and we modeled *θ*, *θ*′, and *ψ* as functions of climate and land cover covariates. In this initial model, we used a finite mixture model on *p* to account for heterogeneity, presumably caused by differences among routes in habitat, observers, bird abundance, and other features that were not explicitly modeled (Clement et al., 2016). We also modeled *p* as a function of climate and land cover covariates and year. Because bird activity often varies with time of day, we also modeled detection as a quadratic function of stop number, which we consider to be a proxy for time of day. From this highly parameterized model, we fit a reduced model by eliminating the covariate with the smallest estimate‐to‐standard error ratio. We continued to eliminate covariates in this way until AIC increased and then selected the model with the lowest AIC. We used this parameter reduction protocol with *ψ*, *θ*, *θ*′, and *p* in turn, maintaining the model structure on *γ* and *ε*. After identifying parsimonious models for *ψ*, *θ*, *θ*′, and *p*, we fit the set of six models for *γ* and *ε* described above. The sequential approach may not yield identical results as a single analysis, but it is a pragmatic approach for dealing with complex models (MacKenzie et al., 2018). By beginning with highly parameterized models and progressing toward reduced models, we reduced the risk of confounding the occupancy and detection processes (MacKenzie et al., 2018). We also checked for potential multi‐collinearity issues by calculating Pearson's correlation coefficient for the different covariates used in the models. We treated an *R* ^2^ < 0.5 as indicative of a lack of multi‐collinearity.

We also evaluated the goodness of fit of the global model for each species using the naïve colonization and extinction rates to calculate the test statistic of a Hosmer–Lemeshow test (Hosmer & Lemeshow, 2000). In this context, the naïve colonization rate is the number of sites that changed from nondetection sites in year *t *to detection sites in year *t* + 1, while the naïve extinction rate is the number of sites that changed from detection sites in year *t *to nondetection sites in year *t* + 1. We divided the routes into deciles based on the expected colonization and extinction rates, and compared the predicted rates to the observed rates with chi‐square tests (*α* = 0.05). If necessary, we combined deciles to ensure that the predicted number of detections in a decile was >4. Although the Hosmer–Lemeshow test statistic is often calculated from predicted presence, vital rates seemed to be more appropriate measures in this case because dynamic models estimate changes in presence, rather than presence (Clement et al., 2016). We note that this use of naïve rates focuses on products of model parameters (i.e., *γ*, *ε*, *p, ψ, ϑ*), and therefore, we do not test the fit of the fully parameterized model. We focused on naïve rates because the true vital rates (i.e., *γ* and *ε*) cannot be directly observed when detection is imperfect, while the naïve rates can be observed.

We used an analysis of deviance approach (ANODEV; e.g., Skalski, 1996) to evaluate the relative importance of climate and land cover to changes in bird distributions. In this procedure, we compared the deviance explained by our parameter of interest to the deviance explained by the most fully parameterized model in the model set:

$$R_{\text{Dev}}^{2}=\frac{\text{Dev}\left(M_{\text{null}}\right)-\text{Dev}\left(M_{\text{cov}}\right)}{\text{Dev}\left(M_{\text{null}}\right)-\text{Dev}\left(M_{\text{full}}\right)}$$

Dev(*M*_null_) is the deviance of Model 3, which accounts for spatial, but not temporal, variation in colonization and extinction probabilities. Dev(*M_f_*_ull_) is the deviance of Model 2, the global model, with both spatial and temporal variation in *γ* and *ε*. We then evaluated the deviance explained by climate and/or land cover by using models 4, 5, and 6 for Dev(*M*_cov_). Higher values of $R_{\text{Dev}}^{2}$ indicate greater explanatory power, with 0 indicating no power, and 1 indicating power equal to that of the fully parameterized model, although $R_{\text{Dev}}^{2}$ could potentially exceed 1 because our covariate models are not strictly nested within Model 2. Therefore, we calculated and report $R_{\text{Dev}}^{2}$ for both climate and land cover. This is not the only possible approach to assessing relative importance of explanatory variables, but it is sound and has been used and recommended by others (Grosbois et al., 2008).

The general ANODEV approach to partitioning variation led us to focus on hypothesis testing as a means of assessing the need for extra model parameters (sources of variation). The small set of specific a priori hypotheses also sets this work apart from exploratory studies investigating the adequacy of many different models. For these reasons, we used likelihood ratio tests as a means of comparing different models, although we note that use of model selection criteria (e.g., AICc) yielded similar inferences.

## 3 RESULTS

Our covariates indicated a small loss of habitat and increased temperatures during the study period, despite the relatively short time scale. For Eastern Wood Pewee, habitat change was >1% on 38% of routes, with the mean share of suitable habitat near routes declining from 41.6% to 40.9%. For Red‐eyed Vireo, habitat change was >1% on 23% of routes, with mean suitable habitat declining from 48.0% to 47.6%. Route temperatures were rarely above the heat stress threshold, but the average time above this threshold per route increased from 0.5 hr per year from 1982 to 1997 to 1.7 hr per year from 1997 to 2012. We also observed a decline in periods of cold stress, from 5,450 to 5,291 hr per route per year. The Pearson's correlation coefficient did not indicate any multi‐collinearity problems among our covariates (R^2^<0.2 in all cases).

### 3.1 Eastern Wood Pewee

For Eastern Wood Pewee, the best‐supported model included both climate and habitat measures as covariates of *ψ*, *θ*, and *θ*′. We excluded climate and habitat covariates from the detection probability model because some of those models did not converge. Therefore, the detection model for each species included year and stop number as covariates. As a result, the global model included 85 parameters (Table 1). The Hosmer–Lemeshow test indicated that the global model fit the data adequately, with no evidence of discrepancies between estimated and observed naïve colonization rates (*χ* ^2^ = 4.41, *df *= 8, *p* = 0.82) and naïve extinction rates (*χ* ^2^ = 11.17, *df *= 8, *p* = 0.19).

**Table 1:** Estimated parameters for correlated‐detection dynamic occupancy Model 2 relating habitat and climate covariates to occupancy dynamics of breeding Eastern Wood Pewee and Red‐eyed Vireo, 1997–2012. Parameter estimates indicate change in log odds of parameters in response to *z*‐transformed covariates. Parameters *ψ*, *ϑ*, *ϑ*′, *γ*, *ε*, *p1*, *p2*, and *π* defined in text

Parameters	Eastern Wood Pewee		Red‐eyed Vireo	
	Estimate	*SE*	Estimate	*SE*
*Ψ* intercept	2.367	0.177	4.136	0.404
*Ψ* habitat	0.556	0.163	2.315	0.300
*Ψ* heat stress	−0.011	0.198	−0.337	0.281
*Ψ* cold stress	0.598	0.166	1.020	0.258
*ϑ* intercept	−1.821	0.026	−1.385	0.010
*ϑ* habitat	−0.055	0.026	0.516	0.013
*ϑ* heat stress	−0.094	0.030	0.017	0.008
*ϑ* cold stress	0.172	0.040	0.258	0.013
*ϑ*′ intercept	2.292	0.045	0.426	0.020
*ϑ*′ habitat	0.316	0.036	1.210	0.018
*ϑ*′ heat stress	−0.006	0.038	0.054	0.013
*ϑ*′ cold stress	−0.622	0.064	0.876	0.019
*γ* intercept	−0.884	0.353	0.010	0.424
*γ* 1999	−0.601	0.585	−0.150	0.532
*γ* 2000	−1.083	0.633	0.516	0.464
*γ* 2001	−0.300	0.484	0.228	0.483
*γ* 2002	−1.113	0.577	0.262	0.511
*γ* 2003	−1.055	0.564	−0.311	0.551
*γ* 2004	−0.656	0.525	−0.179	0.534
*γ* 2005	−0.677	0.533	0.468	0.485
*γ* 2006	−1.014	0.620	−0.217	0.579
*γ* 2007	−0.162	0.453	−0.093	0.548
*γ* 2008	−23.545	70,483.373	−0.478	0.607
*γ* 2009	−0.428	0.455	−0.253	0.566
*γ* 2010	−0.292	0.478	−0.159	0.517
*γ* 2011	−1.136	0.607	0.012	0.490
*γ* 2012	−0.498	0.474	0.141	0.497
*γ* average habitat	0.095	0.091	0.801	0.112
*γ* average heat stress	−0.024	0.067	0.005	0.062
*γ* average cold stress	0.227	0.071	0.358	0.120
*ε* intercept	−3.957	0.386	−3.767	0.242
*ε* 1999	0.365	0.482	−0.189	0.337
*ε* 2000	0.049	0.512	−0.921	0.399
*ε* 2001	−0.266	0.548	−0.532	0.362
*ε* 2002	−0.576	0.660	−1.156	0.398
*ε* 2003	−0.298	0.595	−0.692	0.355
*ε* 2004	0.113	0.509	−0.621	0.343
*ε* 2005	0.173	0.482	−1.096	0.429
*ε* 2006	−0.145	0.561	−1.173	0.463
*ε* 2007	0.451	0.458	−0.894	0.366
*ε* 2008	0.283	0.480	−0.976	0.424
*ε* 2009	0.218	0.496	−0.898	0.381
*ε* 2010	−0.397	0.605	−0.348	0.304
*ε* 2011	0.643	0.455	−0.999	0.406
*ε* 2012	0.153	0.644	−0.803	0.410
*ε* average habitat	0.238	0.090	−1.670	0.122
*ε* average heat stress	−0.071	0.092	−0.075	0.074
*ε* average cold stress	−0.307	0.141	−0.904	0.104
*p1* intercept	−2.172	0.043	1.352	0.038
*p1* 1998	−0.118	0.048	−0.127	0.058
*p1* 1999	−0.112	0.049	−0.043	0.058
*p1* 2000	−0.166	0.050	−0.016	0.059
*p1* 2001	−0.156	0.049	0.031	0.060
*p1* 2002	−0.179	0.049	0.076	0.061
*p1* 2003	−0.216	0.051	0.161	0.064
*p1* 2004	−0.242	0.051	0.066	0.060
*p1* 2005	−0.124	0.050	0.149	0.060
*p1* 2006	−0.182	0.051	0.124	0.059
*p1* 2007	−0.149	0.050	0.184	0.061
*p1* 2008	−0.202	0.052	0.192	0.063
*p1* 2009	−0.221	0.052	0.135	0.061
*p1* 2010	−0.195	0.051	0.062	0.059
*p1* 2011	−0.122	0.051	0.203	0.062
*p1* 2012	−0.227	0.055	0.194	0.062
*p1* stop number	0.033	0.009	−0.055	0.011
*p1* stop number squared	−0.069	0.010	−0.247	0.013
*p2* intercept	−0.921	0.018	−0.557	0.036
*p2* 1998	−0.138	0.033	−0.106	0.050
*p2* 1999	−0.137	0.032	0.000	0.052
*p2* 2000	−0.164	0.032	−0.027	0.051
*p2* 2001	−0.077	0.032	−0.053	0.053
*p2* 2002	−0.101	0.032	−0.019	0.052
*p2* 2003	−0.199	0.033	0.086	0.053
*p2* 2004	−0.186	0.032	0.124	0.057
*p2* 2005	−0.146	0.032	0.076	0.051
*p2* 2006	−0.155	0.032	0.128	0.054
*p2* 2007	−0.153	0.032	0.120	0.054
*p2* 2008	−0.168	0.032	0.072	0.056
*p2* 2009	−0.077	0.033	0.101	0.055
*p2* 2010	−0.114	0.033	0.085	0.060
*p2* 2011	−0.079	0.032	0.124	0.054
*p2* 2012	−0.186	0.033	0.036	0.061
*p2* stop number	0.045	0.007	0.165	0.010
*p2* stop number squared	−0.105	0.008	−0.183	0.010
*π*	0.533	0.064	−0.505	0.065

**Figure 2:**
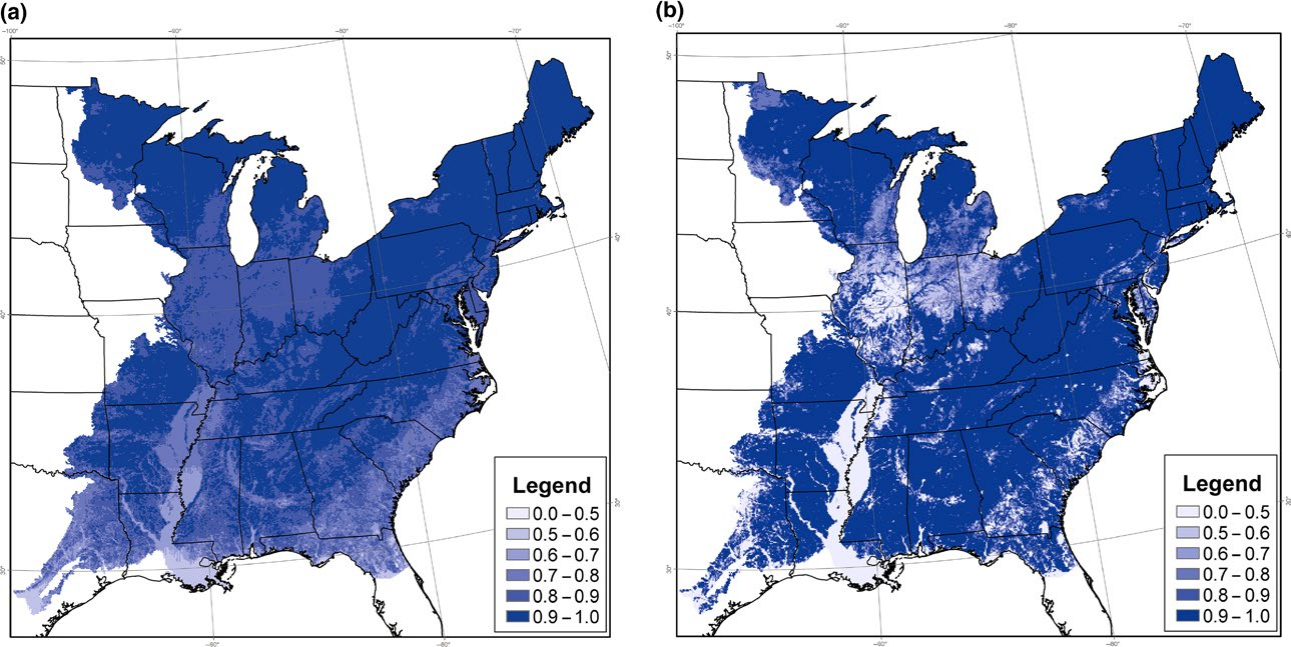
Occupancy probability for (a) Eastern Wood Pewee and (b) Red‐eyed Vireo in the eastern United States, 1997, under Model 2. Occupancy varies by the amount of suitable habitat, hours of heat stress, and hours of cold stress in 1997

**Figure 3:**
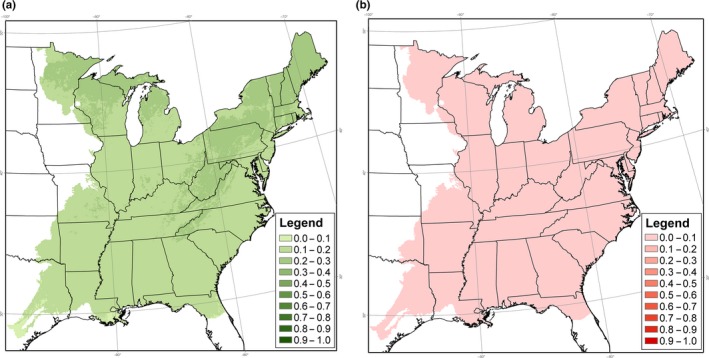
Probability of (a) colonization and (b) extinction for Eastern Wood Pewee in the eastern United States, following 1997, under Model 3. Colonization and extinction vary by the amount of suitable habitat, hours of heat stress, hours of cold stress, all averaged over the 1997–2012 study period

Eastern Wood Pewee were widespread in the study area, with an average probability of occupancy on BBS routes in 1997 of 0.90 in the global model (Figure 2). Occupancy declined slightly to 0.88 by 2012, mirroring the decline in counts reported by the BBS. Initial occupancy was positively related to available habitat and hours of cold stress (Table 1). Probability of detection at the first individual stop during 1997 was low, at 0.13, while probability of detection at the route level was much higher at 0.95 because birds were typically available at many stops within a route. Model 3, which included spatial variation in climate and habitat in the colonization and extinction models, significantly improved model fit, relative to Model 1 (likelihood ratio *χ* ^2^ = 44.19, *df *= 6, *p* < 0.001). Under this model, estimated colonization rates in 1998 ranged among routes from 0.08 to 0.28 (with a mean of 0.18), while extinction rates varied from 0.01 to 0.04 (with a mean of 0.02; Figure 3). Model 2 differed from Model 3 in that it allowed colonization and extinction rates to vary each year. Model 2 improved model fit relative to Model 3, although not significantly due to the number of additional parameters (*χ* ^2^ = 26.79, *df *= 28, *p* = 0.53). Under Model 2, estimated colonization rates varied from 0.00 in 2008 to 0.30 in 1998, while extinction rates varied from 0.01 in 2001–2003 and 2010 to 0.04 in 2011. We used Models 4, 5, and 6 to estimate the percent of improved model fit (measured by the change in deviance) generated by Model 2 that could be accounted for by temporal variation in climate and habitat covariates (Table 2). Temporal variation in habitat accounted for 0.4% of the change in deviance, while temporal variation in climate accounted for 19.6%. Model 6, which accounted for temporal variation in both habitat and climate, indicated that in 1998, colonization in the southwest portion of the study area was above the local average while extinction in the southwest was below the local average, indicating a relatively strong occupancy trend in the southwest in that year (Figure 1). By 2012, this pattern had reversed, yielding a relatively weak occupancy trend in the southwest (Figure 1). None of these models represented a significant improvement over Model 3 (*p* > 0.26). In some cases, the sign of the estimated effect of temporal variation in climate and habitat was opposite of the expected effect, but in no instances were these estimates significant (Table 3).

**Table 2:** $R_{\text{Dev}}^{2}$ for different models of colonization and extinction probabilities for breeding Eastern Wood Pewee and Red‐eyed Vireo, 1997–2012

Model	Covariates on colonization and extinction						Eastern Wood Pewee		Red‐eyed Vireo	
	Intercept	Average Habitat	Average Climate	Habitat Deviation	Climate Deviation	Year Effect	Δ −2LL	$R_{\text{Dev}}^{2}$	Δ −2LL	$R_{\text{Dev}}^{2}$
2	X	X	X			X	0.00	100	0.00	100
6	X	X	X	X	X		−21.38	20.2	−32.30	5.7
4	X	X	X		X		−21.54	19.6	−32.34	5.6
5	X	X	X	X			−26.67	0.4	−34.22	0.1
3	X	X	X				−26.79	0	−34.27	0
1	X						−70.98	NA	−453.11	NA

Note

NA: not applicable.

**Table 3:** Estimated parameters for correlated‐detection dynamic occupancy Model 6 relating habitat and climate covariates to occupancy dynamics of breeding Eastern Wood Pewee and Red‐eyed Vireo, 1997–2012. Parameter estimates indicate change in log odds of parameters in response to *z*‐transformed covariates. Parameters *ψ*, *ϑ*, *ϑ′*, *γ*, *ε*,* p1*, *p2*, and *π* defined in text. For key parameters (bold text), expected signs included

Parameters	Expected	Eastern Wood Pewee		Red‐eyed Vireo	
	Effect	Estimate	*SE*	Estimate	*SE*
*Ψ* intercept		2.367	0.177	4.087	0.381
*Ψ* habitat		0.556	0.163	2.323	0.288
*Ψ* heat stress		−0.011	0.198	−0.322	0.275
*Ψ* cold stress		0.598	0.166	1.054	0.251
*ϑ* intercept		−1.821	0.026	−1.385	0.010
*ϑ* habitat		−0.055	0.026	0.516	0.013
*ϑ* heat stress		−0.094	0.030	0.017	0.008
*ϑ* cold stress		0.172	0.040	0.256	0.013
*ϑ*′ intercept		2.292	0.045	0.426	0.020
*ϑ*′ habitat		0.316	0.036	1.210	0.018
*ϑ*′ heat stress		−0.006	0.038	0.054	0.013
*ϑ*′ cold stress		−0.622	0.064	0.875	0.019
*γ* intercept		−0.884	0.353	0.023	0.162
*γ* habitat deviation	**+**	**−0.292**	**0.478**	**−0.240**	**1.768**
*γ* heat stress deviation	**−**	**−1.136**	**0.607**	**0.064**	**0.194**
*γ* cold stress deviation	**−**	**−0.498**	**0.474**	**2.398**	**2.253**
*γ* average habitat		0.095	0.091	0.788	0.111
*γ* average heat stress		−0.024	0.067	0.004	0.062
*γ* average cold stress		0.227	0.071	0.333	0.119
*ε* intercept		−3.957	0.386	−4.471	0.141
*ε* habitat deviation	**−**	**−0.397**	**0.605**	**0.121**	**1.492**
*ε* heat stress deviation	**+**	**0.643**	**0.455**	**−0.034**	**0.182**
*ε* cold stress deviation	**+**	**0.153**	**0.644**	**2.042**	**2.072**
*ε* average habitat		0.238	0.090	−1.664	0.123
*ε* average heat stress		−0.071	0.092	−0.080	0.074
*ε* average cold stress		−0.307	0.141	−0.939	0.104
*p1* intercept		−2.172	0.043	1.351	0.039
*p1* 1998		−0.118	0.048	−0.128	0.058
*p1* 1999		−0.112	0.049	−0.043	0.058
*p1* 2000		−0.166	0.050	−0.017	0.059
*p1* 2001		−0.156	0.049	0.031	0.061
*p1* 2002		−0.179	0.049	0.076	0.062
*p1* 2003		−0.216	0.051	0.160	0.064
*p1* 2004		−0.242	0.051	0.066	0.061
*p1* 2005		−0.124	0.050	0.149	0.061
*p1* 2006		−0.182	0.051	0.124	0.060
*p1* 2007		−0.149	0.050	0.183	0.062
*p1* 2008		−0.202	0.052	0.191	0.064
*p1* 2009		−0.221	0.052	0.134	0.062
*p1* 2010		−0.195	0.051	0.062	0.060
*p1* 2011		−0.122	0.051	0.202	0.063
*p1* 2012		−0.227	0.055	0.193	0.063
*p1* stop number		0.033	0.009	−0.055	0.011
*p1* stop number squared		−0.069	0.010	−0.247	0.013
*p2* intercept		−0.921	0.018	−0.555	0.037
*p2* 1998		−0.138	0.033	−0.121	0.050
*p2* 1999		−0.137	0.032	−0.025	0.052
*p2* 2000		−0.164	0.032	−0.027	0.051
*p2* 2001		−0.077	0.032	−0.059	0.053
*p2* 2002		−0.101	0.032	−0.012	0.052
*p2* 2003		−0.199	0.033	0.085	0.053
*p2* 2004		−0.186	0.032	0.113	0.059
*p2* 2005		−0.146	0.032	0.082	0.052
*p2* 2006		−0.155	0.032	0.135	0.054
*p2* 2007		−0.153	0.032	0.124	0.055
*p2* 2008		−0.168	0.032	0.073	0.057
*p2* 2009		−0.077	0.033	0.100	0.056
*p2* 2010		−0.114	0.033	0.071	0.062
*p2* 2011		−0.079	0.032	0.120	0.055
*p2* 2012		−0.186	0.033	0.030	0.063
*p2* stop number		0.045	0.007	0.165	0.010
*p2* stop number squared		−0.105	0.008	−0.182	0.011
*π*		0.533	0.064	−0.505	0.065

### 3.2 Red‐eyed Vireo

The best‐supported model for Red‐eyed Vireo included the same covariates as for Eastern Wood Pewee, yielding a global model with 85 parameters (Table 1). The Hosmer–Lemeshow test indicated fit was adequate for the Red‐eyed Vireo global model for both naïve colonization rates (*χ* ^2^ = 5.40, *df *= 4, *p* = 0.25) and naïve extinction rates (*χ* ^2^ = 1.00, *df *= 4, *p* = 0.91).

**Figure 4:**
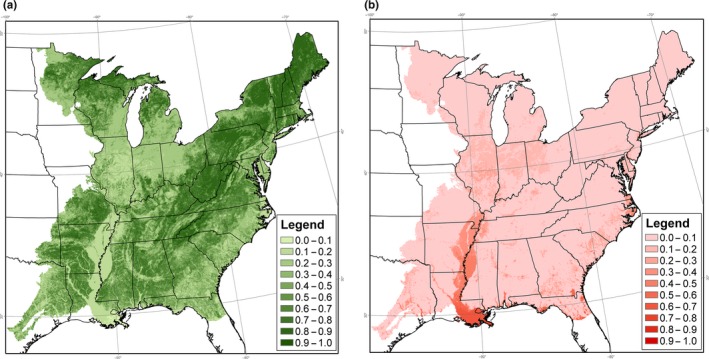
Probability of (a) colonization and (b) extinction for Red‐eyed Vireo in the eastern United States, following 1997, under Model 3. Colonization and extinction vary by the amount of suitable habitat, hours of heat stress, hours of cold stress, all averaged over the 1997–2012 study period

**Figure 5:**
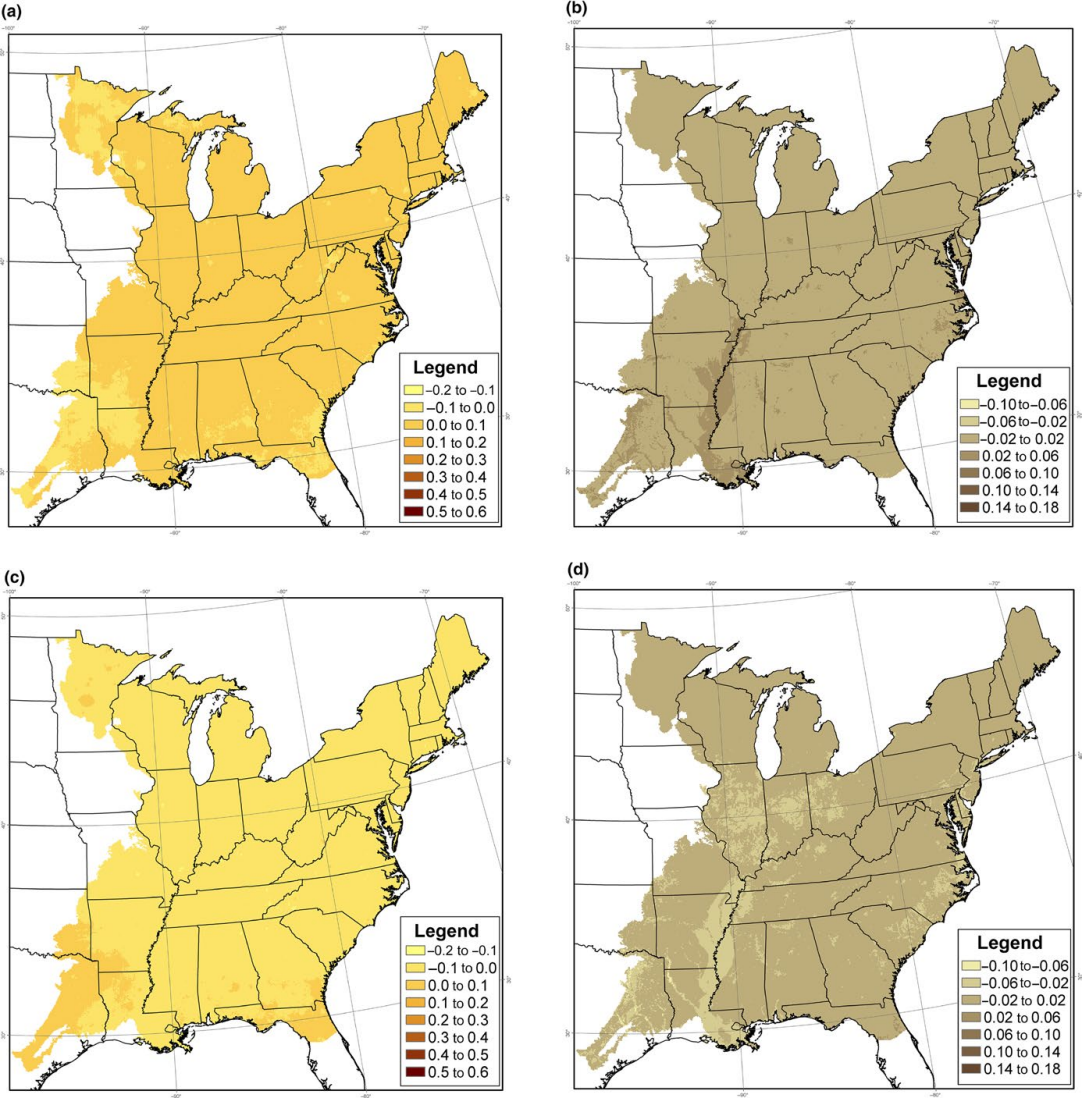
Annual deviation of colonization and extinction rates from average colonization and extinction rates, for Red‐eyed Vireo in the eastern United States, following 1997 and 2011, under Model 6. Deviations in colonization and extinction rates vary with deviations in the amount of suitable habitat, hours of heat stress, hours of cold stress, relative to average levels over the 1997–2012 study period. (a) Colonization deviation following 1997, (b) extinction deviation following 1997, (c) colonization deviation following 2011, and (d) extinction deviation following 2011

Red‐eyed Vireo were similarly widespread in the study area, with an average probability of occupancy of 0.91 in the global model (Figure 2). Occupancy decreased slightly to 0.90 by 2012, in contrast to the increase in counts reported by the BBS. Initial occupancy was positively related to available habitat and hours of cold stress (Table 1). Probability of detection at the first stop was relatively high, at 0.38, while probability of detection at the route level nearly perfect at 0.98. Model 3, which included spatial variation in climate and habitat to the colonization and extinction models, improved model fit dramatically (*χ* ^2^ = 418.84, *df *= 6, *p* < 0.001), relative to the null model. In keeping with the greater change in deviance for Red‐eyed Vireo, Model 3 estimated more spatial variation in vital rates, compared to Eastern Wood Pewee. In 1998, estimated colonization rates varied across BBS routes from 0.10 to 0.88 (mean of 0.49), while extinction rates ranged from 0.00 to 0.48 (mean of 0.04; Figure 4). Model 2, which allowed colonization and extinction rates to vary each year, improved model fit relative to Model 3, but not significantly due to the number of additional parameters (*χ* ^2^ = 34.27, *df *= 28, *p* = 0.19). Under Model 2, estimated colonization rates varied from 0.39 in 2008 to 0.60 in 2000, while estimated extinction rates varied from 0.03 in 2006 to 0.07 in 1998. We used Models 4, 5, and 6 to estimate the percent of the deviance reduction in Model 2 that could be accounted for by temporal variation in climate and habitat covariates (Table 2). We found that temporal variation in habitat accounted for 0.1% of the change in deviance, while climate accounted for 5.6%. Model 6, which accounted for temporal variation in both habitat and climate, indicated that in 1998, both colonization and extinction were above average, indicating higher turnover early in the study period (Figure 5). By 2012, both colonization and extinction rates had declined, indicating greater stability in occupancy (Figure 5). None of these models represented a significant improvement over Model 3 (*p* > 0.74). Some of the estimated effects of temporal variation in climate and habitat differed from expectations, but not significantly (Table 3).

## 4 DISCUSSION

We found that for both bird species, initial occupancy, colonization rates, and extinction rates correlated with spatial variation in climate and habitat covariates. However, colonization and extinction rates were not correlated with temporal variation in climate and habitat covariates. Our results correlating initial occupancy with environmental covariates are, broadly speaking, consistent with biological expectation and a raft of previous studies that have indicated a significant relationship between species distributions and habitat (Robinson, Wilson, & Crick, 2001; Thogmartin, Sauer, & Knutson, 2007), climate (Barbet‐Massin & Jetz, 2014; Bellard, Bertelsmeier, Leadley, Thuiller, & Courchamp, 2012), or both (Seoane, Bustamante, & Díaz‐Delgado, 2004; Sohl, 2014). However, we suggest that estimated relationships between spatial variation in environmental covariates and colonization and extinction rates are of greater ecological interest than relatively phenomenological species distribution models or climate envelope models (Clement et al., 2016). The estimated vital rates of colonization and extinction bring us closer to understanding the dynamic process underlying the distribution of these species. Given constant vital rates, we can also project the equilibrium level of occupancy at sites ($\frac{\gamma_{i}}{\gamma_{i}+\varepsilon_{i}}$, at site *i*). We can compare these projected equilibria to current occupancy to project trends for the species under current environmental conditions. We can also use projected changes in key environmental variables to develop predicted range changes in the future. We argue that these projections are more robust than those from static climate envelope models, which assume that species are currently in equilibrium (Yackulic et al., 2015). In contrast, static models can overestimate range changes when the equilibrium does not hold (Morin & Thuiller, 2009).

Given the importance of global change, we were motivated to investigate factors affecting temporal, as well as spatial, variation in vital rates. In this case, spatial variation must be incorporated into such analyses in order to accommodate the different dynamics expected in different portions of a species range. We view the relationship between temporal variation in covariate values and vital rates as the most relevant to understanding the effect of global change on the recent and future distributions of our study species. Furthermore, a correlation between vital rates and spatially varying covariates may not indicate a similar correlation with temporal changes in those same covariates. For example, a species with strong dispersal constraints might have a strong spatial relationship with a covariate, but a weak temporal relationship due to its immobility (Devictor et al., 2008). Similarly, an estimated spatial relationship may be purely phenomenological due to the nature of spatial variables. When spatial variables follow gradients, it is relatively easy to obtain statistically significant correlations, absent any causal relationship (Grosbois et al., 2008). For example, if colonization rates and temperatures both follow north–south gradients, they may be spatially correlated even if colonization is governed by some other, unidentified factor. In this case, we would expect the weak correlation between temporal changes in temperature and colonization rates to be more indicative of the true relationship than the strong correlation with spatial variation in temperature.

BBS surveys indicate different abundance trajectories for our study species, with counts increasing for Red‐eyed Vireos and decreasing for Eastern Wood Pewees (Sauer et al., 2015). Because abundance and occupancy tend to be linked (Gaston et al., 2000), we expected some divergence in occupancy dynamics. We found that estimated occupancy was high and stable for both species, although there was greater turnover for Red‐eyed Vireos. For example, under Model 3 (spatial variation only in covariates), both average colonization rates (0.49) and average extinction rates (0.04) were at least twice as high for Red‐eyed Vireos, compared to Eastern Wood Pewee (0.18 and 0.02, respectively). Red‐eyed Vireos exhibited greater spatial variation in colonization and extinction rates (Figure 4), although our selected covariates explained little temporal variation for this species. Eastern Wood Pewee colonization and extinction rates exhibited more of a latitudinal gradient, reflecting a spatial correlation with extreme temperature (Figure 3). Our results also hinted at a greater correlation between temporal changes in temperature and occupancy vital rates for Eastern Wood Pewee, which could play a role in the decline of this species, although the results were not significant (Table 3). Alternatively, it has been suggested that Eastern Wood Pewee may be declining due to a loss of forested wintering habitat (Robbins, Sauer, Greenberg, & Droege, 1989) or due to deer‐mediated changes in forest structure on breeding grounds (deCalesta, 1994), illustrating the complexity of ascertaining cause and effect using observational studies in these natural systems.

Although relatively rare (Sirami et al., 2017), other studies have investigated both climate and land cover effects on bird distributions. In agreement with our finding that temporal variation in climate explained much more of the model deviance than temporal variation in habitat, climate‐only species distribution models had better cross‐validation support than habitat‐only models for 409 European bird species (Barbet‐Massin et al., 2012). Similarly, models estimating the number of newly occupied areas by Hooded Warblers (*Wilsonia citrina*) in Ontario using climate data had better information criterion support than models using forest data (Melles, Fortin, Lindsay, & Badzinski, 2011). In contrast, land‐use models outperformed climate models for 18 farmland bird species in the United Kingdom (Eglington & Pearce‐Higgins, 2012). Similarly, vegetation‐based species distribution models had superior performance than climate‐based models for 79 Spanish bird species (Seoane et al., 2004) and for three bird species in New Mexico (Friggens & Finch, 2015). Two studies focused on colonization and extinction rates found mixed results. For Garden Warblers (*Sylvia borin*) in Britain, vegetation explained more variation in colonization, while temperatures explained more variation in local extinction (Mustin, Amar, & Redpath, 2014). For 122 bird species in Ontario, the best‐supported covariates (land cover or climate change) varied among species (Yalcin & Leroux, 2018).

The divergent results among studies regarding the importance of climate and land cover may partly be due to the biology of individual species (Yalcin & Leroux, 2018). For example, farmland birds may be particularly affected by changes in agricultural intensity because of their extensive use of farms (Eglington & Pearce‐Higgins, 2012). Alternatively, it has been argued that spatial scale may play a role in the relative importance of global change factors, with climate more important at large scales (Pearson & Dawson, 2003). We note that two studies that found a larger role for climate both relied on lower‐resolution bird surveys (0.5° atlas in Barbet‐Massin et al., 2012; 10 km^2^ atlas in Melles et al., 2011). In the BBS data that we analyzed, surveys were conducted every 800 m, but we calculated occupancy across each 39.4 km transect. In addition, the cited studies used different data sources and diverse analysis methods, complicating interpretation of the differing results.

While we estimated that climate explained more model deviance than habitat, these estimated relationships were not significant and did not explain a large fraction of variation. There are several potential explanations for why we did not find a substantial response to changes in habitat and climate. First, our data were lacking in dynamic events to inform our models. The covariates changed relatively little over the time period, making it difficult to detect a response. The lack of change was partly because of the relatively short (16‐year) time period studied. Also, we used a moving average for climate data, which tends to reduce differences over short time periods. In addition, the study region has experienced less warming over the past century than other parts of the planet (Walsh et al., 2014). The bird distributions also lacked dynamism. Because extinction rates were low, there were few local extinction events to inform the model. Although colonization rates were high, the fact that initial occupancy was high and extinction rates were low meant there were few opportunities for colonization events.

Second, measurement error may have obscured responses to global change. The PRISM climate data are estimated by interpolation, rather than direct measurement across the study area (Daly et al., 2008). As such, there may be artifacts or anomalies that impeded our estimation and increased uncertainty. Similarly, NLCD data are remotely sensed and may include some measurement error, which can increase uncertainty in our estimates (Homer et al., 2015). Additionally, land cover was only measured in three years, requiring us to impute values in 13 years. As a result, all land cover change that occurred between 2002 and 2006 was recorded in 2002, while changes between 2007 and 2011 were recorded in 2007. This approximation is not ideal, but was necessary given the 5‐year resolution of the data. This lower resolution of the land cover data may have reduced the sensitivity of the model.

Third, we may have misspecified our model. We tried to develop biologically meaningful covariates, but we might not understand the system as well as we hoped. It is possible that bird vital rates respond to climate on a different time scale (i.e., not a 15‐year moving average), or to climate during different times of the year, or to different temperature thresholds, or to different climate components (e.g., precipitation; Illán et al., 2014). It is also possible that the birds respond to finer habitat features than the land cover types we used, or at a different spatial scale (i.e., not 400 m; Pearson & Dawson, 2003). Alternatively, there may be important regional differences or interactions among covariates that we did not capture. Or, it is possible that changes in climate and habitat in their breeding areas simply have little effect on vital rates for these species relative to other factors, such as competition with other bird species, or global change occurring on their wintering grounds in South America (Dormann, 2007). Of course, these misspecification issues are inherent to all observational studies, and not unique to this study.

It would be possible for us to explore some of the misspecification issues just mentioned. For example, we could rerun the same models using new covariates representing a variety of temperature thresholds and habitat buffer sizes to search for a stronger correlation. Experience suggests that persistence would eventually be rewarded with a *p*‐value <0.05 or a low model selection value. However, one reason to avoid such exploration is that it tends to lead to Type I errors, overfit models, and biased parameter estimates (Fieberg & Johnson, 2015). Furthermore, we find the contrast between significant correlations for spatial covariates and nonsignificant correlations for temporal covariates illuminating. In typical climate envelope studies, we would estimate the relationship between covariates and the current distribution of a species, much like our estimate of initial occupancy (Dormann, 2007). Because we obtained significant parameter estimates, we might conclude that we have identified covariates that determine the range of this species. Then, we might use these results to project the future range of the species under various climate scenarios based on the (questionable) assumption that the covariate–species relationship will not change as climate and habitat change (Cuddington et al., 2013; Elith et al., 2010; Gustafson, 2013). In this case, the insignificant correlation with the temporal covariates suggests that our occupancy covariates merely describe the range of these birds, without determining the range. Furthermore, our results suggest that, despite significant results in the static portion of the model, we have yet to identify the most relevant components of climate change shaping the distribution of these birds, although it may be difficult to identify these components given the lack of observed change during a relatively short time period. Finally, our results suggest that projections based on the static portion of our model are unlikely to be reliable, despite their statistical significance.

If our interpretation is correct, it suggests that some projections of future range shifts based on static models rely on unsupported assumptions and are not reliable. We have demonstrated a different approach that does not require the strong assumptions of static modeling approaches. Our approach would be more apt to identify factors that guide species’ response to global change if both the species and the covariates exhibited more dynamism, and if we analyzed a longer time period that encompassed greater change.

## DEDICATION

We dedicate this paper to the late Chandler S. Robbins, “Father” of the North American Breeding Bird Survey (BBS). Data from the BBS form the basis for this specific paper and for hundreds of others focused on topics ranging from local trends to continental macroecology. Chan was a unique combination of bird‐lover and scientist whose vision and inquisitive enthusiasm were an inspiration to all of us fortunate enough to have known him.

## CONFLICT OF INTEREST

None declared.

## AUTHORS’ CONTRIBUTION

JAC and JDN conceived the study. AJT and SW analyzed habitat and climate data. MJC, JEH, and JDN performed the occupancy analysis. MJC led the writing of the manuscript. All authors gave final approval for publication.

## Data Availability

Raw data are available from the following sources. Breeding Bird Survey: https://www.pwrc.usgs.gov/bbs/rawdata/. PRISM climate data: http://www.prism.oregonstate.edu/. NLCD land cover data: https://www.mrlc.gov/. Processed data will be archived with the Dryad Digital Repository.
